# Spatial capture–recapture with multiple noninvasive marks: An application to camera‐trapping data of the European wildcat (*Felis silvestris*) using R package multimark

**DOI:** 10.1002/ece3.6990

**Published:** 2020-12-02

**Authors:** Lea Maronde, Brett T. McClintock, Urs Breitenmoser, Fridolin Zimmermann

**Affiliations:** ^1^ Carnivore Ecology and Wildlife Management KORA Muri bei Bern Switzerland; ^2^ Alaska Fisheries Science Center Marine Mammal Laboratory NOAA‐NMFS Seattle Washington USA

**Keywords:** density, *Felis silvestris*, latent multinomial, monitoring, multimark, partial identity, spatial capture–recapture

## Abstract

In Switzerland, the European wildcat (*Felis silvestris*), a native felid, is protected by national law. In recent decades, the wildcat has slowly returned to much of its original range and may have even expanded into new areas that were not known to be occupied before. For the implementation of efficient conservation actions, reliable information about the status and trend of population size and density is crucial. But so far, only one reliable estimate of density in Switzerland was produced in the northern Swiss Jura Mountains. Wildcats are relatively rare and elusive, but camera trapping has proven to be an effective method for monitoring felids. We developed and tested a monitoring protocol using camera trapping in the northern Jura Mountains (cantons of Bern and Jura) in an area of 100 km^2^. During 60 days, we obtained 105 pictures of phenotypical wildcats of which 98 were suitable for individual identification. We identified 13 individuals from both sides and, additionally, 5 single right‐sided flanks and 3 single left‐sided flanks that could not be matched to unique individuals. We analyzed the camera‐trap data using the R package multimark, which has been extended to include a novel spatial capture–recapture model for encounter histories that include multiple “noninvasive” marks, such as bilaterally asymmetrical left‐ and right‐sided flanks, that can be difficult (or impossible) to reliably match to individuals. Here, we present this model in detail for the first time. Based on a “semi‐complete” data likelihood, the model is less computationally demanding than Bayesian alternatives that rely on a data‐augmented complete data likelihood. The spatially explicit capture–recapture model estimated a wildcat density (95% credible interval) of 26 (17–36) per 100 km^2^ suitable habitat. Our integrated model produced higher abundance and density estimates with improved precision compared to single‐sided analyses, suggesting spatially explicit capture–recapture methods with multiple “noninvasive” marks can improve our ability to monitor wildcat population status.

## Graphical abstract

In this study, we developed and tested a monitoring protocol for the European wildcat using camera trapping. Reliable population estimates as density and abundance are often lacking for this elusive species. We analyzed our camera trap data using the open‐source R package multimark, which has been extended to include a novel spatial capture–recapture model.

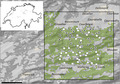

## 1 INTRODUCTION

Conservation and management require reliable information about population size, density, structure, and trends over time (Williams et al., 2002). Here, we focus on the European wildcat (*Felis silvestris*) that was once widespread throughout Europe before a drastic decline of the population during the 19^th^ century, mainly caused by persecution and habitat loss (Schauenberg, 1970). In Switzerland, the species has been protected by national law since 1962. In the last three decades, it has slowly returned to its former distribution range in the Jura Mountains and, recently, even expanded to the Swiss Plateau and the Alps (Zimmermann et al., 2017, 2018; KORA, unpublished data). However, the European wildcat is still listed as an endangered species of high national priority in Switzerland (BAFU, 2019).

The monitoring of wildcats is particularly challenging due to their elusive nature (e.g., they are nocturnal, prefer dense cover, and occur at low densities), resulting in a low detection rate (Anile et al., 2012; Kilshaw et al., 2015). In addition, they can be confused with tabby domestic cats (*Felis catus*) when only observed briefly. To tackle these issues, noninvasive techniques, such as DNA hair sampling and camera trapping, are increasingly used. A widely applied method to monitor wildcats is the so‐called lure stick approach (Hupe & Simon, 2007), using wooden sticks sprayed with valerian tincture. Valerian is known to attract wildcats and evoke rubbing behavior, and lure sticks can thus be used to collect hair samples for genetic analyses (Hupe & Simon, 2007; Steyer et al., 2013). This method can be applied to assess wildcat distribution and the degree of hybridization with domestic cats (Steyer et al., 2016). However, the use of genotyped hair samples for capture–recapture analyses to calculate population parameters, such as abundance and density, requires a relatively large number of genotyped samples (Kéry et al., 2011) and hence is rather expensive (Wening et al., 2019). Moreover, there are suspicions that lure sticks with valerian may be a less reliable method to detect wildcats compared to camera traps as not all individuals are interested in valerian and thus do not show rubbing behavior (Velli et al., 2015).

Here, we assess an alternative monitoring approach based on camera trapping. The use of camera traps (CTs) to study population size of species with distinctive natural marks has become an important tool for monitoring rare or cryptic species in a wide range of environments (Zimmermann & Foresti, 2016). Wildcats can be distinguished from domestic cats on good quality camera trap images based on several distinct pelage characters (Anile et al., 2012; Gil‐Sánchez et al., 2020; Kilshaw et al., 2015; Maronde et al., 2020; Müller, 2011a; Velli et al., 2015), and individual wildcats can be identified according to their own unique pelage patterns (Müller, 2011b; Wening et al., 2019;). So far, few studies have applied capture–recapture techniques to estimate wildcat population densities from CT surveys (Anile et al., 2012; Can et al., 2011; Kilshaw et al., 2015).

Being able to identify an individual based only on a photograph depends on the distinctiveness of natural or artificial marks. As both flanks of a wildcat differ, an individual must be photographed bilaterally at least once in order to reliably match the flanks. Although two out of three CT studies estimating wildcat density by means of capture–recapture methods considered this requirement and used two‐camera settings, that is, one at each side of the track, it is unlikely that simultaneous pictures can be gathered for all individuals encountered during a survey (e.g., the present study).

To date, researchers were therefore forced to either analyze each flank separately or to match flanks based on questionable or untestable assumptions (Anile et al., 2012; Kilshaw et al., 2015). Neither approach is ideal, as they are prone to loss of precision and bias due to data loss or violations of assumptions, especially for the sparse datasets common to these studies (McClintock et al., 2013). Only recently, new statistical methods aiming to resolve this issue have been developed (Augustine et al., 2018; Bonner & Holmberg, 2013; McClintock et al., 2013). Using Bayesian methods, the R package multimark (McClintock, 2015) allows practitioners to combine capture–recapture (CR) data arising from multiple “noninvasive” marks in closed (Otis et al., 1978) or open (e.g., Cormack‐Jolly‐Seber; Lebreton et al., 1992) population models. Also referred to as “partial identity” models (Augustine et al., 2018), these methods were originally developed for estimating population abundance or survival from bilateral photo‐identification records (where left‐ and right‐hand flanks are considered as two different types of natural marks), but multimark can also be applied to other types of marks (e.g., based on photograph and DNA records) or conventional mark–recapture data consisting of a single mark type. In May 2016 (multimark version 2.0.0, and higher), a new version extending the package to spatially explicit capture–recapture models (SCR) was released. In addition to facilitating estimation of population density, SCR models have the advantage that they can account for individual heterogeneity in detection probability that is attributable to the spatial proximity of individual activity centers to trap locations. Detection probability can also be affected by behavioral responses, such as trap shyness (e.g., due to the flash of the CT) or trap happiness (e.g., due to attractants). Furthermore, the probability of detection can vary by season, habitat type, and other spatio‐temporal factors. Such models can be easily specified in multimark using simple linear model formulas already familiar to most R users, and the package also includes model selection and multimodel inference capabilities based on Barker and Link (2013). Until now, the SCR model in multimark has to our knowledge only been applied once to calculate population density estimates of the Persian leopard *Panthera pardus saxicolor* (Farhadinia et al., 2019).

In this study, we aimed at developing and testing a camera‐trapping protocol for estimating wildcat density by means of the new partial identity spatial capture–recapture model implemented in multimark. This SCR model is presented here in detail for the first time. We estimated density for all habitats within the study area and for only suitable wildcat habitat based on a habitat suitability model. We additionally compared the results from the partial identity models with those from conventional analyses based on only right‐ or left‐sided encounters.

## 2 MATERIALS AND METHODS

### 2.1 Study area

**Figure 1:**
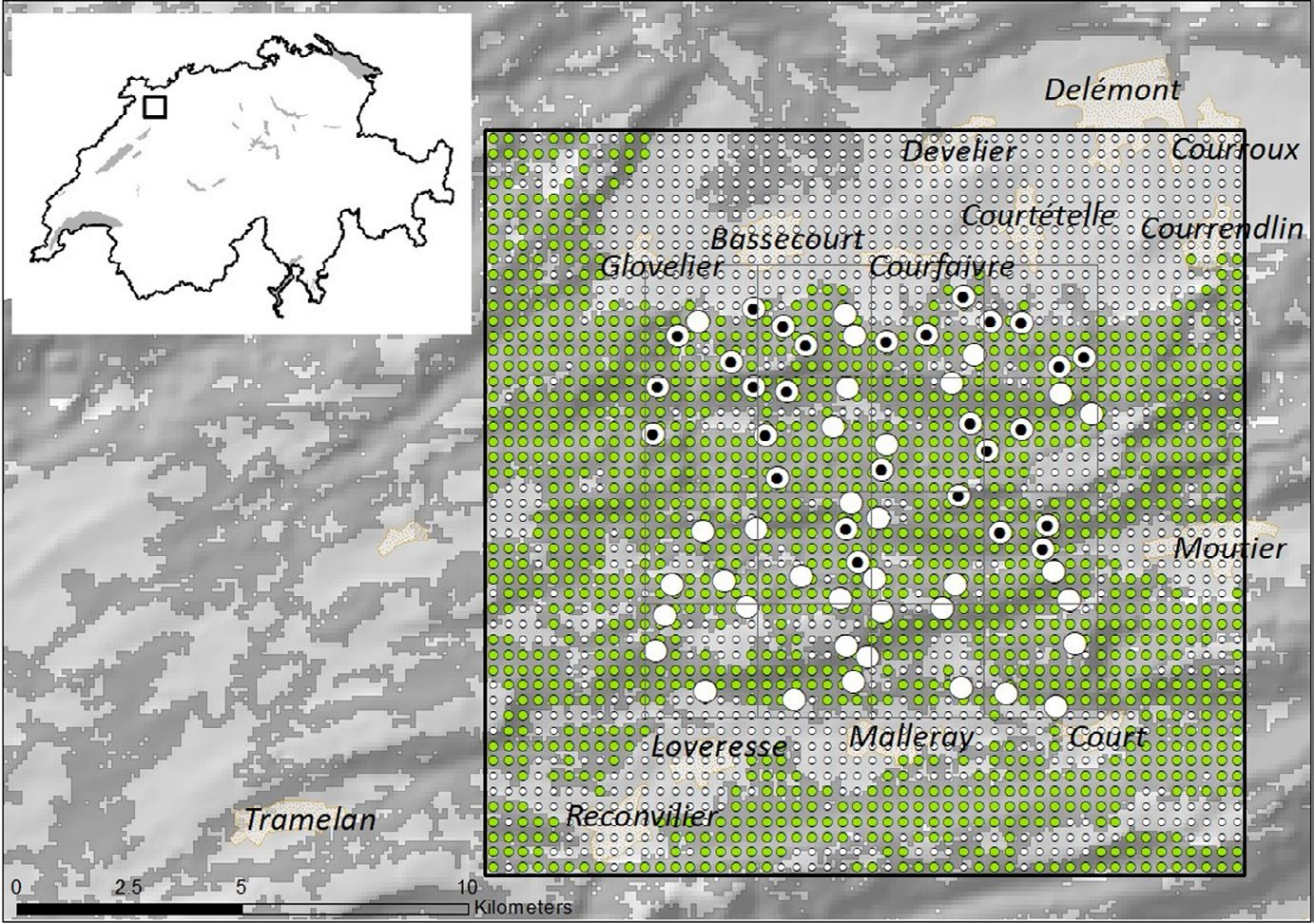
Study area in the northern Swiss Jura Mountains. The white circles show the camera trap sites and those with inset black dots indicate wildcat detections. The small green and white dots of the state space show the potential 121 m spaced activity centers, within and outside the suitable habitat, respectively. The inset shows the location of the state space in Switzerland (square)

The study took place in the northern Swiss Jura Mountains, in the Cantons of Jura and Bern. The Jura Mountains are a secondary limestone mountain chain with half of the entire surface covered by forest. The 10 × 10‐km study area consisting of 16 2.5. × 2.5 km grid cells was located south–west of the city of Delémont and west of the city of Moutier (47°17′ N, 7°15′ E) (Figure 1). Elevation ranges from 460 to 1,337 m above sea level. Population density in the study area ranges from about 54 inhabitants in the administrative district Perrefitte (Bern) to about 97 inhabitants/km^2^ in Haute‐Sorne (Jura) (www.bfs.admin.ch, 2019).

The exact position of the camera grid was chosen for its high proportion of connected forest areas (~70%), the absence of larger human settlements or other anthropogenic structures, and evidence for the presence of wildcats from previous lynx monitoring surveys (KORA unpublished data). The presence of wildcats was further confirmed by the study of Weber et al. (2010) and by the local game wardens. According to a habitat model for the European wildcat in Switzerland (Weber, 2018), about 75% of the study area was considered suitable habitat for the wildcat (Figure 1).

### 2.2 Sampling design and site selection

The size of the study area and the CT density was based on other wildcat CT studies (Anile et al., 2012; Can et al., 2011; Kilshaw et al., 2015), home range sizes from published literature, and telemetry studies in Switzerland (Breitenmoser et al., 2009, unpublished report). Wildcat home ranges can vary strongly, with females having on average much smaller home ranges than males (females 3 km^2^ and males 12 km^2^; e.g., Germain et al., 2008, Klar, Fernandez, et al., 2008, 2008, Götz et al., 2018). The smallest home ranges of female wildcats recorded are about 2 km^2^ (reviewed in Götz et al., 2018). An important requirement of conventional capture–recapture models but not mandatory in spatially explicit models is that each individual must have a probability > 0 of being detected, and thus, there should be at least one sampling site per smallest female home range (usually the smallest home range in the population) (Karanth & Nichols, 1998). We therefore chose a density of one CT site per 1.56 km^2^. We chose a camera grid of 100 km^2^, which we believed to be large enough to obtain a sufficient sample size (at least 20 individuals) for reliable parameter estimation based on nonspatial capture–recapture models. In total, 64 CT stations were placed in the 10 × 10‐km grid (4 sites per 2.5 × 2.5 km). Optimal CT sites were selected based on our own experience and those of the local game wardens. All sites were chosen in the forest along forest roads, hiking trails and, more rarely, game trails, often close to the forest edge while ensuring to cover the whole elevation gradient.

### 2.3 Timing of the CT session

CTs were active for 60 nights, from 25 February to 25 April 2016. We have chosen the late winter as study period, because this time is the mating period of wildcats. It is assumed that this period is particularly suitable as the wildcats wander a lot in search of a partner and are most likely to react to the attractant valerian at this time (Hupe & Simon, 2007; Kéry et al., 2011).

### 2.4 CTs, lure sticks, and site maintenance

At each station, two Xenon white flash CTs (Cuddeback Digital, Green Bay, USA, models Ambush, Capture or C1) were deployed in different combinations. The units were set to operate over 24 hr. On each side of the track, we placed one CT, but not exactly opposite to the other one to avoid overexposure of pictures by the flash. The CTs were installed approximately 40 to 60 centimeters above ground level to obtain photographs of wildcats in the best position for individual identification. Opposite of one of the CTs, we placed a lure stick, that is, a small wooden stick of about 60 cm height, sprayed with valerian tincture (Hänseler, Herisau, Switzerland). The attractant was used to entice wildcats to walk in front of the CTs and to linger in front of it and hence be subjected to multiple exposures, which aids identification of the species and individuals. Furthermore, by rubbing at the stick with coarse surface, wildcats leave a hair sample, which can be used for genetic analyses. We collected all detected hair samples during the survey. Sites were checked on average every seven days in order to replace batteries and memory cards, to collect hair samples, and to refresh the valerian lure.

### 2.5 Identification of the species and individuals

All cat pictures obtained were first categorized as domestic cat or wildcat pictures according to the following key pelage characteristics (Kitchener et al., 2005; Ragni & Possenti, 1996): (a) shape of the tail, (b) number and shape of tail rings, (c) black tail tip, (d) dorsal line until the base of the tail, (e) two shoulder stripes, and (f) four to five neck stripes. More details on the species’ evaluation procedure can be found in: [Bestimmungshilfe zur Unterscheidung von Wild‐ und Hauskatzen anhand von Fotofallenbildern aus dem Schweizer Jura“, Maronde et al., 2020)]. We cannot completely rule out that hybrids were included in one or both groups since the identification of hybrids according to the phenotype is difficult or not possible (Krüger et al., 2009; Ballesteros‐Duperon et al., 2015, but see Kitchener et al., 2005), However, no recent hybridization events have been detected in the genetic samples collected in the study area (Tobias Reiners and Carsten Nowak, Senckenberg Research Institute, pers. comm.).

For individual identification, we carefully evaluated all pictures of wildcats to identify individual marks consisting of typical characteristics in coat pattern, such as shape and position of stripes and spots, the number and shape of tails rings, and other individual marks. Individual identification was conducted by at least two people. In a first step, one experienced person examined all pictures of wildcats and assigned a picture to either an individual of which both flanks are known or to a single flank. A second person checked each assessment of the first observer. In case of a disagreement, a third person was consulted. If no agreement had been found after this procedure, the picture would have been excluded from any further analysis. However, this was never the case in our study.

### 2.6 Statistical Background

Spatial capture–recapture data are typically represented by a collection of encounter histories for the n unique individuals encountered across J traps, $Y=\left\{Y_{1},Y_{2},\cdots ,Y_{J}\right\}$, where $Y_{j}=\left\{y_{1j},y_{2j},\cdots ,y_{\mathrm{nj}}\right\}$ and each element of $y_{\mathrm{ij}}=\left(y_{ij1},y_{ij2},\cdots ,y_{\mathrm{ijT}}\right)$ indicates whether or not individual i was detected $\left(y_{\mathrm{ijt}}=1\right)$ or not detected $\left(y_{\mathrm{ijt}}=0\right)$ at trap j on each of t=1,⋯,T sampling occasions. When there are two marks types and it is difficult (or impossible) to reliably match individuals, then both Y and n are unknown. Instead, we have $\tilde{Y}=\left\{{\tilde{Y}}_{1},{\tilde{Y}}_{2}\right\}$, where ${\tilde{Y}}_{m}=\left\{{\tilde{Y}}_{m_{1}},{\tilde{Y}}_{m_{2}},\cdots ,{\tilde{Y}}_{m_{J}}\right\}$ for $m\in \left\{1,2\right\}$, ${\tilde{Y}}_{m_{j}}=\left\{{{\tilde{y}}_{m}}_{1j},{{\tilde{y}}_{m}}_{2j},\cdots ,{{\tilde{y}}_{m}}_{n_{m}j}\right\}$, $n_{m}$ is the number of unique individuals encountered for mark type *m*, and each element of ${{\tilde{y}}_{m}}_{\mathrm{ij}}=\left({{\tilde{y}}_{m}}_{ij1},{{\tilde{y}}_{m}}_{ij2},\cdots ,{{\tilde{y}}_{m}}_{\mathrm{ijT}}\right)$ indicates whether or not individual i was detected $\left({{\tilde{y}}_{m}}_{\mathrm{ijt}}=m\right)$ or not detected $\left({{\tilde{y}}_{m}}_{\mathrm{ijt}}=0\right)$. Depending on the mark types and sampling design, sometimes some subset of the encounter histories can be matched with certainty; in this case, we have $\tilde{Y}=\left\{{\tilde{Y}}_{\mathrm{known}}{,\tilde{Y}}_{1},{\tilde{Y}}_{2}\right\}$ where ${\tilde{Y}}_{\mathrm{known}}$ consists of the $n_{\mathrm{known}}$ individuals for which the true encounter histories are known with certainty. Using extensions of the methodology originally proposed by Bonner and Holmberg (2013) and McClintock et al. (2013), R package multimark (McClintock, 2015) facilitates the joint analysis of type 1 $\left({\tilde{Y}}_{1}\right)$, type 2 $\left({\tilde{Y}}_{2}\right)$, and known $\left({\tilde{Y}}_{\mathrm{known}}\right)$ encounter histories while accounting for uncertainty in n. Here, we describe for the first time in detail the spatial capture–recapture extension of the multiple mark closed population abundance (or density) estimator in multimark.

As in the nonspatial multiple mark models of Bonner and Holmberg (2013) and McClintock et al. (2013), we assume there are five possible types of encounters with two mark types: $y_{\mathrm{ijt}}=0$ indicates nondetection, $y_{\mathrm{ijt}}=1$ indicates detection of mark type 1 (e.g., left‐flank photograph), $y_{\mathrm{ijt}}=2$ indicates detection of mark type 2 (e.g., right flank photograph), $y_{\mathrm{ijt}}=3$ indicates a nonsimultaneous detection of both mark types (e.g., separate left‐ and right flank photographs), and $y_{\mathrm{ijt}}=4$ indicates simultaneous detection of both mark types (e.g., both left‐ and right flanks photographed simultaneously by the two CTs placed at a given site). The Bayesian implementation in multimark is similar in spirit to the spatial capture–recapture model of Royle et al. (2009), but, as with all models in multimark, it relies on a “semicomplete” data likelihood (King et al., 2016) instead of a data‐augmented complete data likelihood. As in Royle et al. (2009), the detection probability for individual *i* at trap *j* and time *t* $\left(p_{\mathrm{ijt}}\right)$ can be modeled as a function of distance to the latent individual “activity centers” as well as time‐ or trap‐dependent covariates using a complementary log–log link:

$$\mathrm{cloglog}\left(p_{\mathrm{ijt}}\right)=x_{\mathrm{jt}}\beta +\log\left(g_{\mathrm{ij}}\right),$$

where $x_{\mathrm{jt}}$ is a row vector of length *K* containing the covariates for trap j at time t, β is a column vector of corresponding coefficients, $g_{\mathrm{ij}}$ is some function of distance from trap j to the latent activity center for individual i, and $\mathrm{cloglog}\left(argument\right)=\log\left(‐\log\left(1‐argument\right)\right)$. A common choice that is available in multimark is the half‐normal detection function:

$$g_{\mathrm{ij}}=\exp\left(‐\frac{d_{\mathrm{ij}}^{2}}{2\sigma^{2}}\right),$$

where $d_{\mathrm{ij}}=\|l_{j}‐s_{i}\|$ is the Euclidean distance between the location of trap $j\;\left(l_{j}\in S\right)$ and activity center $s_{i}\in S$, and S is the state space of the point process (i.e., the study area encompassing the trapping array). An exponential detection function is also available. The state space has to be chosen large enough so that no wildcat individual outside of this area has any probability of being photographed by a CT in the array. In our wildcat application, S was defined by using a $2.5\hat{\sigma }$ buffer around the trapping array, where $\hat{\sigma }=\sqrt{2}$ was chosen based on a preliminary analysis for model *M_c_* (see Model fitting and multimodel inference).

A SCR model gives inferences about two different scales of habitat use: first, where the wildcats are in general (this is characterized by the location of the activity center); and second, where they are at any particular point in time relative to the activity center (this is characterized by the parameter σ^2^). The movement parameter σ^2^ represents thus the space use of the wildcats about their activity centers. By assuming a bivariate normal model for detection, the estimated movement parameter can be converted into a 95% home range radius (e.g., Repucci et al., 2011). The script in R is the following: (σ^2^)*(qchisq(0.95,2)^0.5).

### 2.7 Data analysis

Capture–recapture data are commonly summarized in the form of encounter histories. In our case, a sampling occasion is defined as three consecutive time frames of 24 hr starting at noon, which results in a total of 20 sampling occasions for the duration of the study. Within any sampling occasion, only one detection event of each individual at a given site was considered in the analyses. A single detection event comprises all photographs of an individual wildcat at a given site within a time frame of 30 min. We assume that the population was closed due to the relatively short duration of our survey and because the study period did not encompass the main period of birth and dispersal.

For a given set of latent encounter histories $\left(Y\right)$ that could have generated the observed encounter histories $\left(\tilde{Y}\right)$, the semicomplete data likelihood for the spatial closed population abundance estimator in multimark is:

$$\left[Y,s,z|\beta ,\sigma ,\delta ,\alpha ,N\right]\propto \frac{1}{{\left(p^{*}\right)}^{n}}\prod_{i=1}^{\tilde{n}}\prod_{j=1}^{J}\prod_{t=1}^{T}\pi_{ijt}\times \frac{N!}{\left(N‐n\right)!}{\left(p^{*}\right)}^{n}{\left(1‐p^{*}\right)}^{N‐n},$$

where

$$\pi_{\mathrm{ijt}}=\left\{\begin{array}{cc} 1‐m_{\mathrm{jt}}z_{i}p_{\mathrm{ijt}} & \text{if}\;y_{\mathrm{ijt}}=0\\ m_{\mathrm{jt}}p_{\mathrm{ijt}}\delta_{1} & \text{if}\;y_{\mathrm{ijt}}=1\\ m_{\mathrm{jt}}p_{\mathrm{ijt}}\delta_{2} & \text{if}\;y_{\mathrm{ijt}}=2\\ m_{\mathrm{jt}}p_{\mathrm{ijt}}\left(1‐\delta_{1}‐\delta_{2}\right)\left(1‐\alpha \right) & \text{if}\;y_{\mathrm{ijt}}=3\\ m_{\mathrm{jt}}p_{\mathrm{ijt}}\left(1‐\delta_{1}‐\delta_{2}\right)\alpha & \text{if}\;y_{\mathrm{ijt}}=4 \end{array}\right\},$$

$s=\left(s_{1},s_{2},\ldots ,s_{\tilde{n}}\right)$, N is the population size, $p^{*}=1‐\int_{S}^{}\prod_{j=1}^{J}\prod_{t=1}^{T}\left(1‐m_{\mathrm{jt}}p_{\mathrm{ijt}}\right)ds_{i}$ is the probability any given individual is detected at least once, $m_{\mathrm{jt}}=1$ if trap j was operational at time t ($m_{\mathrm{jt}}=0$ otherwise), $\delta_{1}$ is the (conditional) probability of a type 1 encounter, $\delta_{2}$ is the (conditional) probability of a type 2 encounter, α is the (conditional) probability of a simultaneous type 1 and 2 encounter, and $\tilde{n}=n_{\mathrm{known}}+n_{1}+n_{2}$. For the latent $z=\left(z_{1},z_{2},\ldots ,z_{\tilde{n}}\right)$, we assume $z_{i}|\psi ∼\text{Bernoulli}\left(\psi \right)$, where ψ is the probability that a randomly selected individual from the $\tilde{n}$ observed individuals belongs to the n unique individuals encountered at least once (i.e., $n=\sum_{i=1}^{\tilde{n}}z_{i}$). Similar to King et al. (2016), we approximate $p^{*}$ by summing over a fine spatial grid composed of Q cells:

$$p^{*}\approx 1‐\frac{\sum_{q=1}^{Q}\prod_{j=1}^{J}\prod_{t=1}^{T}\left(1‐m_{\mathrm{jt}}h_{q}p_{\mathrm{qjt}}\right)}{\sum_{q=1}^{Q}h_{q}},$$

where $s_{q}\in S$ is the centroid of grid cell q, and $h_{q}=1$ if cell q contains suitable habitat ($h_{q}=0$ otherwise). In our wildcat application, we used Q=2450 such that grid cells were approximately 0.11 km^2^. We provided two density estimates: 1) without differentiating between suitable and unsuitable wildcat habitat (i.e., $h_{q}=1$ for q=1,…,Q); and 2) considering only suitable wildcat habitat. For the habitat mask, we used a previously developed habitat suitability model for the European wildcat in Switzerland (Weber, 2018) and excluded nonhabitat such as towns and intensive agricultural areas (Figure 1).

The resulting posterior distribution for our Bayesian model is:

$$\begin{array}{cc} & \left[Y,s,z,\beta ,\sigma ,\delta ,\alpha ,N,\psi |\tilde{Y}]\propto [Y,s,z|\beta ,\sigma ,\delta ,\alpha ,N][\tilde{Y}|Y\right]\\ & \;\times [s][z|\psi ][\beta ][\sigma ][\delta ][N][\psi ], \end{array}$$

where $\left[\tilde{Y}|Y\right]=1$ if $\tilde{Y}$ could have been spawned from Y (otherwise $\left[\tilde{Y}|Y\right]=0$), $\left[s\right]=\prod_{i=1}^{\tilde{n}}\left[s_{i}\right]$, $\left[s_{i}\right]=1/\sum_{q=1}^{Q}h_{q}$ is a joint discrete uniform distribution over the coordinates of the centroids of the $\sum_{q=1}^{Q}h_{q}$ grid cells that contain suitable habitat, $\left[z|\psi \right]=\prod_{i=1}^{\tilde{n}}\left[z_{i}|\psi \right]$, $\left[\beta \right]=\prod_{k=1}^{K}\left[\beta_{k}\right]$, $\beta_{k}∼N\left(\mu_{\beta_{k}},\sigma_{\beta_{k}}^{2}\right)$, $\sigma ∼\text{Unif}\left(a_{0}^{\sigma },b_{0}^{\sigma }\right)$ for $0<a_{0}^{\sigma }<b_{0}^{\sigma }$, $\delta =\left(\delta_{1},\delta_{2},1‐\delta_{1}‐\delta_{2}\right)∼\text{Dirichlet}\left(a_{0}^{\delta },b_{0}^{\delta },c_{0}^{\delta }\right)$, $\alpha ∼\text{Beta}\left(a_{0}^{\alpha },b_{0}^{\alpha }\right)$, $\left[N\right]\propto 1/N$, and $\psi ∼\text{Beta}\left(a_{0}^{\psi },b_{0}^{\psi }\right)$. All prior distribution hyperparameters can be user‐specified in multimark (see package dumentation for default values). In our wildcat analysis, we specified $\mu_{\beta_{k}}=0,\sigma_{\beta_{k}}^{2}=1.75,a_{0}^{\sigma }=10m,b_{0}^{\sigma }=16550m$, and $a_{0}^{\delta }=b_{0}^{\delta }=c_{0}^{\delta }=a_{0}^{\alpha }=b_{0}^{\alpha }=a_{0}^{\psi }=b_{0}^{\psi }=1$. We also assumed $\delta_{1}=\delta_{2}$ because we had no reason to suspect these conditional probabilities should be different based on the study design, and preliminary analyses confirmed there was no detectable difference in the conditional probability of a right‐ or left‐sided detection.

### 2.8 Model fitting and multimodel inference

As detailed in Appendix S1 of McClintock (2015), multimark accounts for uncertainty in n by using a Markov chain Monte Carlo algorithm to integrate over the set of possible Y that could have generated the observed $\tilde{Y}$. While the mathematical and computational details are perhaps of little interest to ecologists, multimark performs these operations in the background and requires only simple data formatting and model specification formulas familiar to most R users. We specified four models for the baseline encounter probability: model *M*_0_ including constant detection probability; model *M*_e_ including trap elevation effects (as wildcats tend to prefer areas with shorter snow cover durations, their detection probability might decrease with increasing elevation); model *M*_c_ including a behavioral response to the first capture; and model *M*_e+c_ including additive elevation and behavioral effects.

For each of the four models (*M*_0_, *M*_e_, *M*_c_, *M*_e+c_), we generated five Markov chains of 320,000 iterations (10,000 adaptations and burn‐in of 20,000 iterations) using the “*multimarkClosedSCR”* function in multimark. For each model, the Gelman–Rubin–Brooks multivariate potential scale reduction factor was 1.0 across all chains, and all monitored parameters had effective sample sizes >4,000. We performed Bayesian multimodel inference by drawing 500,000 iterations from the 5 chains for each model using the reversible jump MCMC algorithm described by Barker and Link (2013) and implemented in the “*multimodelClosedSCR*” function in multimark using the default equal prior model weights.

To analyze right‐ and left‐sided data separately, we used the “*markClosedSCR*” function in multimark. We specified the same four models for the baseline encounter probability and again used the “*multimodelClosedSCR*” function for multimodel inference. For more details on these functions, see also McClintock et al. (2020).

## 3 RESULTS

During the entire session, at least one of the two CTs per site was always active; hence, 100% of the available 3,840 (60 nights × 64 CT sites) trap nights were realized. During the 60 nights, we obtained 105 pictures of phenotypical wildcats during 75 events. The capture success rate was 1.95 detection events/100 trap nights. Seven pictures of 6 events were not suitable for individual identification; these were discarded from further analyses. The remaining 98 pictures were summarized in triads which resulted in 64 detection events. Wildcats were detected at 28 different CT sites out of 64 located in the northern part of the study area (Figure 1).

### 3.1 Multiple mark analysis

We were able to match some single flanks of wildcats after the 60 nights due to additional information gained after the survey from camera trapping. Altogether, we could identify 13 individuals from both sides (${\tilde{Y}}_{\mathrm{known}}$) and, additionally, 5 single right‐sided flanks and 3 single left‐sided flanks which could not be merged with any of the 13 individuals.

**Figure 2:**
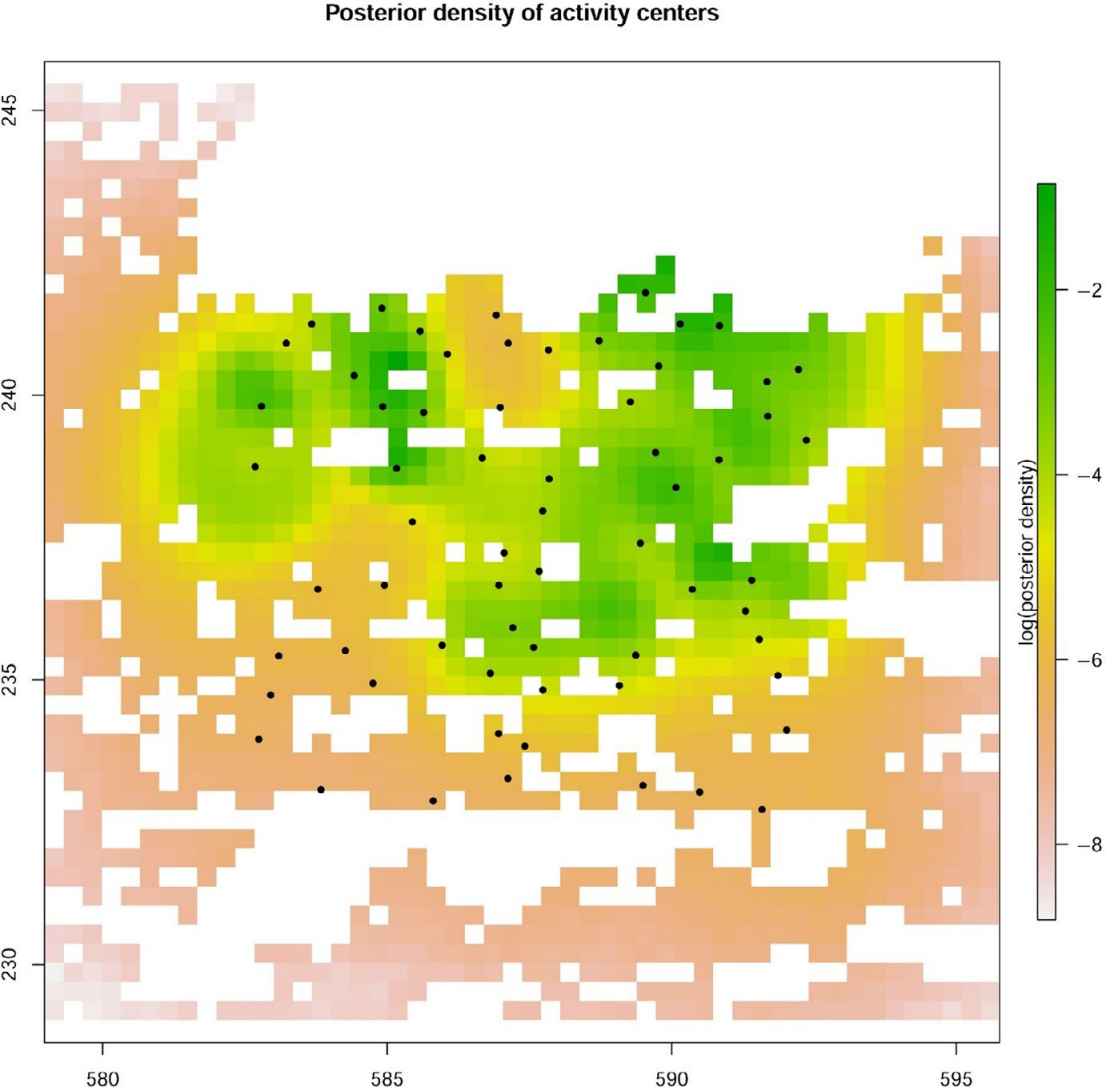
Logarithm of the model‐averaged posterior density of wildcats relative to the trapping array (black dots) resulting from the integrated analysis, considering only suitable wildcat habitat. Each pixel is marked with $\log\left(E\left[N\left(q\right)|\text{data}\right]\right)$ where $N\;\left(q\right)$ is the number of activity centers $\left(s\right)$ within grid cell*q*. X and Y‐axes indicate the coordinates of the Swiss Grid in kilometers

For suitable habitat only, model *M*_e+c_ accounted for 0.30 of the posterior model weights, while *M*_0_ represented 0.27 and *M*_c_ *and M_e_* both 0.21 of the posterior model weights. The model‐averaged estimate of population density (95% credible interval) was 26 (17 – 36) per 100 km^2^ suitable habitat (Figure 2), and the model‐averaged movement parameter σ^2^ was 0.87 (0.34–2.45) km. This translates under the assumption of a bivariate normal movement model into a 95% home range radius of 2.13 km and a home range area of 14.3 km^2^ (see Table 1 in Appendix S2 for further model parameters).

When considering all habitats as being equal (i.e., without differentiating between suitable and unsuitable habitat), the model *M*_e_ accounted for 0.33 of the posterior model weight, while *M*_0,_ *M*_e+c,_ *and M*_c_ represented 0.27, 0.23_,_ and 0.17 of the posterior model weight. The model‐averaged posterior population density was 17 (11 – 25) individuals per 100 km^2^_,_ and movement parameter σ^2^ was 0.74 (0.35 –2.26) km, translating into a 95% home range radius of 1.81 km and a home range area of 10.3 km^2^ (see Table 2 in Appendix S2 for further model parameters).

### 3.2 Single mark analyses

**Figure 3:**
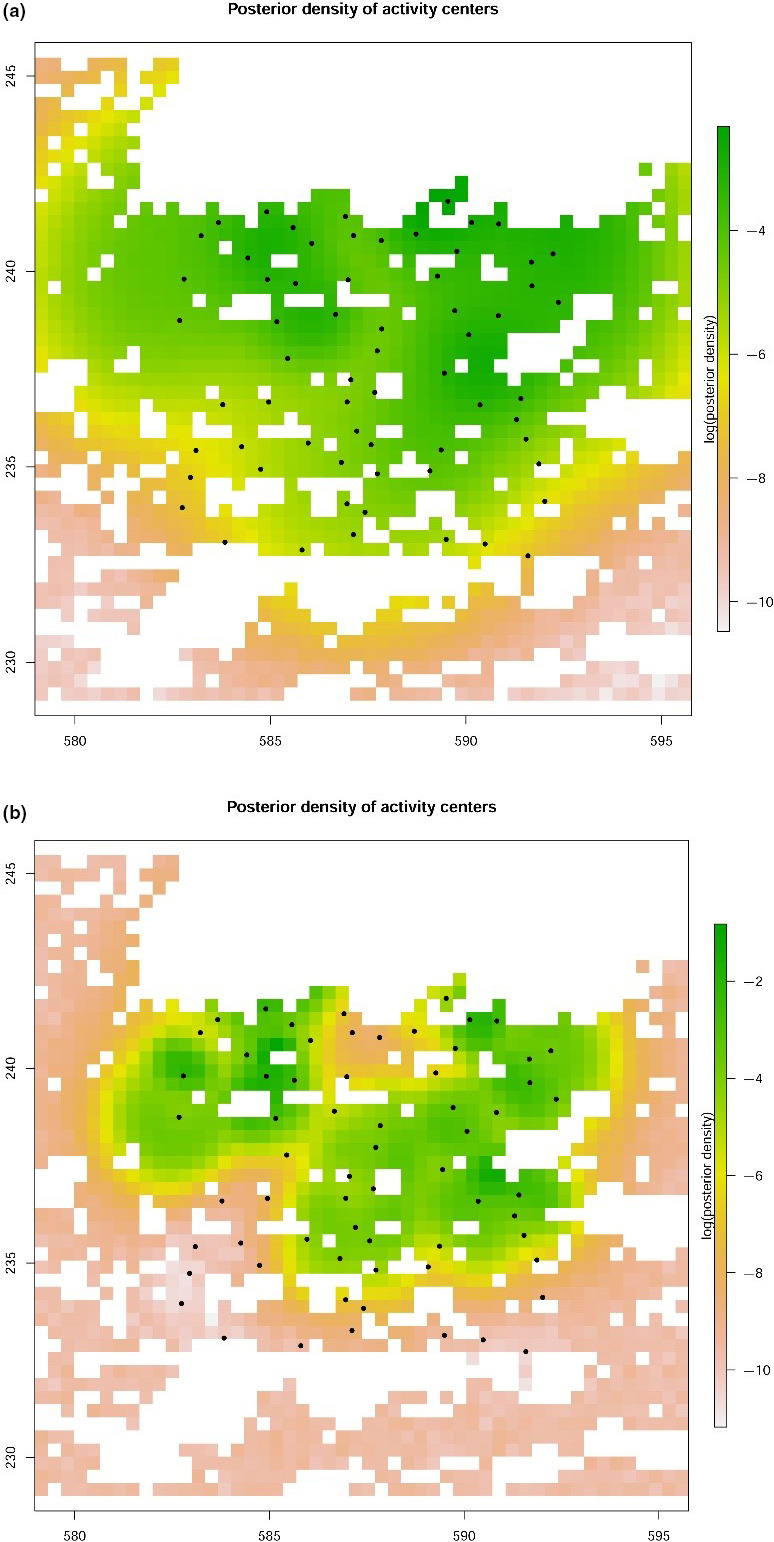
Logarithm of the model‐averaged posterior density of wildcats relative to the trapping array (black dots) resulting from the analysis of the right (a) and left flanks (b). Each pixel is marked with $\log\left(E\left[N\left(q\right)|\text{data}\right]\right)$ where $N\left(q\right)$ is the number of activity centers $\left(s\right)$ within grid cell *q*. X‐ and Y‐axes indicate the coordinates of the Swiss Grid in kilometers

We identified 16 right‐sided flanks and 15 left‐sided flanks. For right flanks, the model *M*_e+c_ accounted for 0.51 of the posterior model weight, and *M*_c,_ *M*_e,_ *and M*_0_ accounted for 0.45, 0.02_,_ and 0.01 of the posterior model weight, respectively. The model‐averaged estimate of population density was 20 (13 − 31) per 100 km^2^ suitable habitat (Figure 3), and the movement parameter σ^2^ was 3.11 (1.63 – 5.95) km, translating into a 95% home range radius of 7.61 km and a home range area of 181.9 km^2^ (see Table 3 in Appendix S1 for further model parameters). For left flanks, the model *M*_e_ accounted for 0.47 of the posterior model weight, and *M*_0,_ *M*_e+c,_ and *M*_c_ accounted for 0.25, 0.18, and 0.10 of the posterior model weight. The model‐averaged estimate of population density was 23 (14–34) per 100 km^2^ suitable habitat (Figure 3), and the movement parameter σ^2^ was 0.49 (0.28 – 1.44) km translating into a 95% home range radius of 1.20 km and a home range area of 4.5 km^2^ (see Appendix S2, Table 4 for further model parameters).

## 4 DISCUSSION

In this study, we developed a camera‐trapping monitoring protocol for European wildcat density estimation in a 10x10 km study area in the northern Jura Mountains. The capture success rate in our study was 1.95 detection events per 100 trap nights, which is within the range of other camera‐trapping studies (Kilshaw et al. 2015: 1.4; Can et al., 2009: 1.8; Velli et al., 2015: 3.1; Anile et al., 2014: 6.48/detection events per 100 trap nights). We had to discard only seven out of 105 pictures (6.7%) for individual identification due to an insufficient quality. We believe that it would be much higher if we would have used CT with IR flash. Hence, for further studies aiming at estimating wildcat densities, we recommend using xenon white flash CTs, which produce better color images both night and day and hence facilitate identification of both species and individuals compared to CTs with IR flash.

Although we set two CTs per site and used valerian as lure, we could not identify both flanks of all individuals detected during the survey. This can happen for several reasons: If a wildcat is moving across the central line of the paired CTs field of view, it may not always be detected by one or both of the paired CTs if any step of the trigger, registration, and image quality process fails (see Findlay et al., 2020 for further details). Most modern CTs rely on a passive infrared sensor and are only triggered when the temperature differential between the target and the background is greater than 2.7°C (Meek et al., 2012). Malfunctions of CTs can also occur, for example, due to empty batteries. Alternatively, the animal could only move across the detection zone of one of the paired CTs and being registered by it, as usually they are not positioned exactly opposite to each other to prevent the flash of the opposing CT causing overexposure of the image. Hence, we chose the R package multimark and implemented a SCR model with partial identity to jointly analyze the left‐ and right‐sided data without having to rely on untestable assumptions about how to attribute the pictures to individual cats.

Our results showed that the study design was suitable for generating a sufficient amount of data for calculating population estimates of reasonable precision. Although density estimates resulting from the integrated, left‐ and right‐sided analyses only slightly differed in this particular case, the integrated analysis nevertheless produced higher density estimates and improved precision. Moreover, the estimated movement parameter σ^2^ was considerably smaller in the integrated analysis compared to the right flank analysis (i.e., the density is more concentrated around the estimated activity centers in the integrated surface map; Figures 2, 3). The home range estimates for the integrated model are within the range of wildcat home range sizes in Central Europe. However, the model for the right flanks gives home range estimates which are unrealistically high.

This highlights the utility of integrated analyses, which will often both reduce bias and improve precision (Augustine et al., 2018; McClintock et al., 2013). The estimated density of 26 (17 – 36) individuals per 100 km^2^ suitable wildcat habitat is within the range of results from other studies in western central Europe. According to the published literature, wildcat densities range between 10 and 50 individuals per 100 km^2^ (Germany: 20–50 per 100 km^2^, for example, Götz & Roth, 2007, Knapp et al., 2002; France: 10 to 50 per 100 km^2^ (Stahl, 1986)). Most of these values originate from telemetry studies and used different methodological approaches. Only a few studies have applied spatial capture–recapture modeling to estimate wildcat densities, and Anile et al. (2014) calculated a wildcat density of 32 ± *SD* 10 wildcats per 100 km^2^ on Etna Volcano (Sicily, Italy) by means of CT, using the R package secr. Kilshaw et al. (2015) conducted a survey on Scottish wild living cats and used the R package SPACECAP to generate a density estimate of 68.2 ± SE 9.5 individuals/100 km^2^. However, this cannot be considered as a representative estimate of wildcat density, as hybrids were included in the survey, and the hybridization rate is known to be extremely high (up to 100%) in Scotland (Breitenmoser et al., 2019). In Switzerland, Kéry et al. (2011) estimated wildcat density at 29 (95% CI: 19–42) individuals per 100 km^2^ for a study area in the Blauen Range of the Jura Mountains in the Basel District by means of genetic capture–recapture.

Direct comparisons with previous studies need to be drawn with caution not only because of the different methodology applied, but also because, to our knowledge, none of the other studies integrated a suitable habitat mask in the analysis. Values are therefore not directly comparable because the proportion of suitable habitat in the state space could differ between the studies. Our results highlight that inferred wildcat density in a fragmented landscape can vary considerably between “all habitats” and “suitable habitat” only. Our estimate almost doubled after potential activity centers located in nonhabitat were excluded from the state space. Hence, when the state space includes unsuitable habitat, we recommend investigators to report density estimates per unit of suitable habitat to facilitate comparisons to other study areas which may exhibit different proportions of suitable wildcat habitat.

In our study, we did not detect any wildcats in the southern part of our study area in about 35 km^2^ of the 100 km^2^ trap array (Figure 1). This resulted in lower posterior density estimates in the south (Figures 2, 3). Possible reasons for the absence of wildcat detections in the southern part of the study area, which may include competitive exclusion by domestic cats or a lower habitat suitability, will be investigated further in another study.

Male wildcats have larger home ranges than females (Götz et al., 2018; Klar, Fernandez, et al., 2008, 2008), which leads to heterogeneity in the SCR model parameter estimates (notably σ). Sexual dimorphism is not strong in wildcats, and body size cannot be assessed based on pictures alone. Furthermore, the wildcats’ relatively inconspicuous genitals which are generally covered by the brushed tail did not allow us to sex the individuals. Females are mostly recognized as such when photographed with their cubs, but females were already alone during the study period. If this type of detection heterogeneity is ignored in capture–recapture models, density can be underestimated (Mohamed et al., 2019); therefore, our density estimates could be biased low and should perhaps be considered as conservative. Sex can be determined by means of noninvasive genetic sampling, but, in our study, the sex could only be determined for 7 individuals (2 females and 5 males; all known from both sides). While the analysis could have been split by sex if this were known for all individuals encountered, this information could not be included as a covariate in our analyses because these integrated models have yet to be extended to accommodate individual‐level covariates.

In our study, we used a valerian‐treated lure stick to collect hairs for subsequent genetic analyses and to keep the wildcat in front of the CT as long as possible to collect good reference pictures of both flanks to link the genetic profile to the phenotype. Valerian is known to have an appealing effect on wildcats and may trigger some individuals to revisit the site. In general, it is not recommended to use attractants in form of lure or bait in CR studies, since the deployment of any kind of attractant can introduce a change in the behavior of an individual in response to the first encounter event (Otis et al., 1978). Here, we accounted for this potential bias by including the behavioral model in the analyses. In the integrated model, we did not find strong evidence of a behavioral response to first capture. The right flank analysis suggests there is strong evidence of a "trap happy" behavior effect (0.96 of all posterior model weight). There is some evidence of "trap happy" behavior effect also in the left‐side analyses, but it is not very strong based on posterior model weights. For an effect of elevation, we also only found little evidence. There is a very weak trend that the baseline probability of detection decreases with elevation (see also Appendix S1: Figure 1). A potential explanation for this trend is that during winter, wildcats avoid areas with heavy snow cover and therefore use low altitude areas more predominantly during this period (Mermod & Liberek, 2002). The low level of effects is not surprising given the relatively low number of (re)‐captures.

In addition, attractants can also amplify individual heterogeneity in encounter probability as they might have variable effects on animals depending on age, sex, or social status (for further disadvantages of the use of attractants in capture–recapture studies see Zimmermann & Foresti, 2016). It is assumed that males have a stronger response to valerian than females, and a bias toward hair samples from male individuals (up to 70% percent) has been observed in wildcats (Steyer et al., 2016). This could potentially have induced bias in our density estimators (Gerber et al., 2012, Garotte et al., 2012) as we could not include sex as covariate in our model. Given the multiple aspects of using an attractant, advantages and disadvantages should be carefully counterbalanced with regard to the study objectives. As wildcats regularly patrol along forest roads, we believe that the detection rate of wildcats without using attractant would be high enough for SCR analyses. For example, high numbers of wildcat detections were recorded during an opportunistic camera‐trapping survey for wolves in the southern Jura Mountains without attractants (KORA, unpublished data). Furthermore, wildcat density could be biased if hybrids were included in the density estimates. Hence, for making corrections, the hybridization rate should be monitored on a regular basis. In the present study, however, no corrections were made as no recent hybridization events have been detected.

Here, we provided a framework for wildcat density estimations by means of camera trapping using a new spatially explicit capture–recapture model for partial identity in R package multimark. In addition to combining left‐ and right‐sided images for partially identified individuals, identity information from other data sources, such as physical captures during a telemetry project or images from subsequent CT studies, can be integrated into capture histories. The population estimates obtained by using integrated models like multimark can reduce bias and improve precision compared to traditional single‐sided analyses. Moreover, since SCR models in multimark rely on a “semicomplete” data likelihood (King et al., 2016) and are written in the C programming language, models can be fitted faster and more efficiently than alternative Bayesian approaches (typically coded in native R or BUGS/JAGS) that rely on data augmentation and complete data likelihoods (e.g., Augustine et al., 2018; Gopalaswamy et al., 2012; Royle et al., 2009). To our knowledge, multimark is also the fastest and most flexible user‐friendly capture–recapture software available for Bayesian multimodel inference. For future studies focusing on wildcat density estimation, we recommend investigators follow our approach as it enables the full use of the available data and produces reliable wildcat density estimates, which are crucial for monitoring population trends and informing management policies for effective wildcat conservation.

## CONFLICT OF INTEREST

None declared.

## AUTHOR CONTRIBUTION

**Lea Maronde:** Conceptualization (equal); Data curation (lead); Formal analysis (equal); Methodology (equal); Writing‐original draft (lead); Writing‐review & editing (equal). **Brett McClintock:** Formal analysis (equal); Methodology (equal); Writing‐original draft (equal); Writing‐review & editing (supporting). **Urs Breitenmoser:** Funding acquisition (lead); Writing‐original draft (equal). **Fridolin Zimmermann:** Conceptualization (equal); Formal analysis (equal); Methodology (equal); Writing‐original draft (equal); Writing‐review & editing (equal).

## Supporting information

Appendix S1

Appendix S2

## Data Availability

Wildcat pictures are available at https://www.koracenter.ch. R scripts and input data for the integrated model for only suitable habitat are provided at https://datadryad.org/stash/share/ikb_BFpGFOz1tTJlsbiI_4QX58‐ASypyrBDmxvoO8LE.
