# Supplementary figures and images for: Spatial capture–recapture with multiple noninvasive marks: An application to camera‐trapping data of the European wildcat (Felis silvestris) using R package multimark
Source: Ecol Evol. 2020 Dec 2;10(24):13968–79. doi: 10.1002/ece3.6990 (PMC7771165; doi:10.1002/ece3.6990)

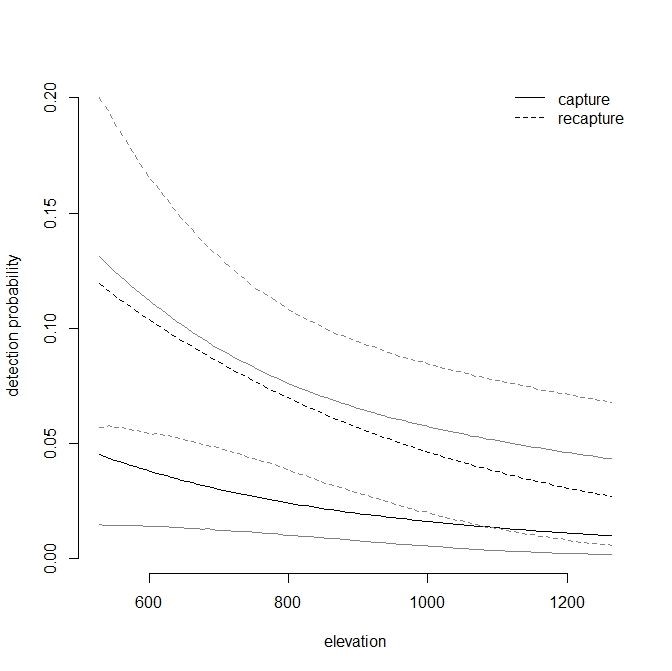

Supplement: Supplementary file 1 — Appendix S1 [file ECE3-10-13968-s001.jpg]
