# Supplementary material for: Spatial capture–recapture with multiple noninvasive marks: An application to camera‐trapping data of the European wildcat (Felis silvestris) using R package multimark
Source: Ecol Evol. 2020 Dec 2;10(24):13968–79. doi: 10.1002/ece3.6990 (PMC7771165; doi:10.1002/ece3.6990)
Supplement: Supplementary file 2 — Appendix S2 [file ECE3-10-13968-s002.docx]

**Appendix 2**

Table 1: Model parameters for the integrated analysis including only suitable habitat. In brackets the posterior model weights. Posterior mean an 95% credible intervals (CI). D= population density per km^2^, N= abundance estimate, $\alpha$ = (conditional) probability of a simultaneous type 1 and 2 encounter, β (c) =behavioral effect to first capture, β (e) =effect of elevation, σ^2^ = distance term for the detection function (half-normal detection function), δ = (conditional) probability of a type 1 encounter or of a type 2 encounter, ψ = the probability that a randomly selected individual from the $n ̃$ observed individuals belongs to the $n$ unique individuals encountered at least once.

| Parameter | Mean (95%CI) |
| --- | --- |
| ***Model M0 (0.27)*** | |
| *D* | 0.26 (0.18-0.36) |
| *N* | 41.09 (29-57) |
| *α* | 0.94 (0.80-1.00) |
| *δ* | 0.37 (0.32-0.42) |
| *σ^2^* | 0.46 (0.32-0.65) |
| *ψ* | 0.87 (0.71-0.97) |
| ***Model Mc (0.21)*** | |
| *D* | 0.25 (0.17-0.36) |
| *N* | 40.32 (28.00-58.00) |
| *α* | 0.94 (0.81-1.00) |
| β (c) | 0.71 (0.22-1.75) |
| *δ* | 0.37 (0.32-0.42) |
| *σ^2^* | 0.97 (0.35-2.47) |
| *ψ* | 0.87 (0.71-0.97) |
| ***Model Me (0.21)*** | |
| *D* | 0.27 (0.19-0.37) |
| *N* | 42.78 (30.00-60.00) |
| *Α* | 0.94 (0.81-1.00) |
| β (e) | -0.34 (-0.70-0.01) |
| *δ* | 0.37 (0.32-0.42) |
| *σ2* | 0.49 (0.34-0.75) |
| *ψ* | 0.88 (0.71-0.98) |
| ***Model Me+c (0.30)*** | |
| *D* | 0.25 (0.17-0.36) |
| N | 39.70 (27.00-58.00) |
| α | 0.94 (0.81-1.00) |
| *β (c)* | 0.93 (0.13-1.78) |
| *β (e)* | -0.37 (-0.71--0.04) |
| *δ* | 0.37 (0.32-0.42) |
| *σ^2^* | 1.43 (0.39-2.80) |
| *ψ* | 0.88 (0.71-0.98) |
